# Cardiac computed tomography in patients with symptomatic new-onset atrial fibrillation, rule-out acute coronary syndrome, but with intermediate pretest probability for coronary artery disease admitted to a chest pain unit

**DOI:** 10.1186/s40001-018-0303-3

**Published:** 2018-01-24

**Authors:** Matthias Koopmann, Liane Hinrichs, Jan Olligs, Michael Lichtenberg, Lars Eckardt, Dirk Böse, Stefan Möhlenkamp, Johannes Waltenberger, Frank Breuckmann

**Affiliations:** 1 Division of Electrophysiology, Department of Cardiovascular Medicine, University of Münster, Münster, Germany; 2 Department of Anesthesiology, Arnsberg Medical Center, Arnsberg, Germany; 3 Department of Angiology, Arnsberg Medical Center, Arnsberg, Germany; 4 Department of Cardiology, Arnsberg Medical Center, Stolte Ley 5, 59759 Arnsberg, Germany; 5 Clinic of Cardiology and Intensive Care Medicine, Bethanien Hospital Moers, Moers, Germany; 6 Department of Cardiovascular Medicine, University of Münster, Münster, Germany

**Keywords:** Cardiac computed tomography, Atrial fibrillation, Coronary artery disease, Chest pain unit, Intermediate pretest probability

## Abstract

**Background:**

Atrial fibrillation (AF) and coronary artery disease (CAD) may be encountered coincidently in a large portion of patients. However, data on coronary artery calcium burden in such patients are lacking. Thus, we sought to determine the value of cardiac computed tomography (CCT) in patients presenting with new-onset AF associated with an intermediate pretest probability for CAD admitted to a chest pain unit (CPU).

**Methods:**

Calcium scores (CS) of 73 new-onset, symptomatic AF subjects without typical clinical, electrocardiographic, or laboratory signs of acute coronary syndrome (ACS) admitted to our CPU were analyzed. In addition, results from computed tomography angiography (CTA) were related to coronary angiography findings whenever available.

**Results:**

Calcium scores of zero were found in 25%. Median Agatston score was 77 (interquartile range: 1–270) with gender- and territory-specific dispersal. CS scores above average were present in about 50%, high (> 400)-to-very high (> 1000) CS scores were found in 22%. Overall percentile ranking showed a relative accordance to the reference percentile distribution. Additional CTA was performed in 47%, revealing stenoses in 12%. Coronary angiography was performed in 22% and resulted in coronary intervention or surgical revascularization in 7%. On univariate analysis, CS > 50th percentile failed to serve as an independent determinant of significant stenosis during catheterization.

**Conclusions:**

Within a CPU setting, relevant CAD was excluded or confirmed in almost 50%, the latter with a high proportion of coronary angiographies and subsequent coronary interventions, underlining the diagnostic value of CCT in symptomatic, non-ACS, new-onset AF patients when admitted to a CPU.

## Background

Acute chest pain with potentially life-threatening conditions is commonly encountered in emergency departments, and the need for prompt and accurate diagnosis has led to the establishment of chest pain units (CPUs) [1]. Within the CPU processes, efforts in optimizing patients’ care also address to the low-risk troponin-negative subset, including the establishment of noninvasive diagnostic work-up in selected patients. Still, coronary artery disease (CAD) leading to myocardial ischemia is the most common cause for admission to a CPU. However, CPUs are often also supposed to take care of patients with the first onset of atrial fibrillation (AF), especially when associated with symptoms suggestive of ischemia [2]. As much as 25% of patients with CAD also exhibit AF; conversely, AF occurs in 2–21% of patients with ACS [3, 4]. Determining pretest risk assessment of coronary artery calcification (CAC) for risk stratification in CAD has been a subject of intensive investigation. CAC scanning has emerged as a robust predictor for coronary events in subjects with an intermediate pretest probability for CAD [5, 6]. A higher CAC burden carries a greater risk for future CAD events and all-cause mortality, thereby appropriately reclassifying a majority of patients into low- and high-risk categories. The Agatston score has been developed as an attempt to reflect a marker for the quantity and location of CAC within the coronary artery circulation, and has attracted a considerable body of research and clinical attention with further imaging improvements over the recent years [5, 7, 8].

Registry data revealed that cardiac computed tomography (CCT) is currently underrepresented in the diagnostic work-up in certified CPUs in Germany despite its high negative predictive power [9]. The present update of the certification criteria now points toward the benefit of cardiac CT angiography, but still provides no mandatory rules for its use in selected patients [10]. So far, data on CAC burden in patients with the first diagnosed, symptomatic AF and an intermediate pretest probability for CAD are missing. The aim of our study was to analyze the clinical value of CCT in the aforementioned patient population within the CPU concept.

## Methods

We reviewed our CCT imaging database and retrospectively included patients who presented during a 2-year-period (from October 2012 to September 2014) to the CPU at our institution. By institutional CPU protocol, CCT was performed in case of the new-onset AF associated with acute atypical chest pain and an intermediate pretest probability for coronary stenosis; if otherwise, coronary angiography should have been used and in case of low-to-intermediate pretest probability for coronary stenosis if ergometry (as the local stress test of first choice) remained nondiagnostic or could not be performed due to various reasons [11]. Atypical chest pain was defined as nonanginal thoracic discomfort not precipitated by physical exertion or emotion. AF was defined according to the current ESC guidelines for the management of AF [12, 13]. Major exclusion criteria were initial triage as acute coronary syndrome (ACS), clinical presentation with typical angina pectoris, preexisting CAD, preexisting AF, new diagnostic ischemic changes on the initial ECG, initial troponin exceeding the 99th percentile of the local assay with a relevant dynamic rise or fall suggestive for ischemia, and positive stress test indicating reversible ischemia. Within our clinical setting, there were no given exclusion criteria for the assessment of CAC except for patients’ refusal. Exclusion criteria for CCT angiography comprised: pregnancy, renal failure with a glomerular filtration rate below 45 ml/min/1.73 m^2^ and known allergy on contrast agents. Patients with overt or subclinical hyperthyroidism as well as patients with valvular AF were also excluded. At the time of image acquisition, all eligible patients had sinus rhythm following spontaneous, medical, or electrical cardioversion.

### CCT imaging

Cardiac computed tomography scans were performed using a 128-slice CT scanner (Aquilion CX, Toshiba Medical Systems, Tokyo, Japan).

### Calcium scoring (CS)

CTs were acquired in a single-slice axial scan from the base to the apex with an image acquisition time of 350 ms. A slice thickness of 3 mm was chosen. Prospective ECG triggering was performed at 80% of the R–R interval. The CAC score was determined according to the methods of Agatston et al. [7] and expressed in Hounsfield units. At least four contiguous pixels with a CT density of ≥ 130 Hounsfield units were used to define an area of CAC. Total CAC scores comprising all calcified coronary lesions as well as regional CAC scores for left main (LM), left anterior descending artery (LAD), left circumflex artery (LCX) and right coronary artery (RCA) were computed. Percentile ranking was performed on the basis of the German population-based Heinz Nixdorf Recall study [14]. Binary subgroup analysis were done for patients with CS = 0 versus CS > 0. In addition, descriptive analysis comprised patients over the 50th percentile declared as above average.

### Computed tomography angiography (CTA)

By protocol, patients with a CS of > 0 and < 400 were assessed by additional CTA. In case of diffuse calcifications of the entire coronary tree, selected patients with a CS of slightly beyond 400 may also have been triaged to subsequent CTA according to the clinical decision made by the attending physician. Image acquisition was performed with a collimation of 2 × 64 × 0.5 mm and a reconstruction interval of 0.3 mm (scanner settings: 120 kv and dynamic 350–450 mA, gantry rotation time: 350 ms, helical pitch: 34.2, pitch factor: 0.53). 80 ml of contrast medium (Iomeron 400 mg/ml, Bracco Imaging, Milan, Italy) were injected at 4.5 ml/s followed by 30 ml saline. The scan was initiated according to the bolus-tracking technique. Metoprolol (standard dosage: 5 mg) was intravenously administered before CTA with a titration dose in patients with heart rate > 70 bpm. Prospective ECG-triggered reconstruction was favored over retrospective ECG-gated reconstruction using automated best cardiac phase algorithms whenever a stable and low heart rate was available (heart beat variability < 15 bpm during breath-hold exam). Coronary arteries were divided into 15 segments according to the American Heart Association classification [15]. Moderate and severe stenoses were judged as significant stenosis (> 50% lumen diameter) and considered as obstructive.

### CS and CTA

All scans were performed in supine position in mid-inspiration breath-hold. The reconstructed CT image data were transferred to the computer workstation for post-processing. All analyses were performed by a single experienced reader (FB) using a Vitrea^®^ workstation (Toshiba Medical Systems, Tokyo, Japan).

### Coronary angiography

The decision for or against further invasive regimen was made by clinical decision pathways by the attending interventional cardiologist on the case. High CS > 400 or obstruction in the CTA favored coronary angiography during index stay. Because of the retrospective character, the decision to initiate invasive diagnostics was independent from the current analysis.

### Statistical analysis

Descriptive data are reported as median and interquartile range (IQR) for continuous variables and count and percentage for categorical variables. The Wilcoxon rank sum test was used to compare continuous variables. Categorical variables were analyzed using Fisher’s exact test or Kruskal–Wallis rank sum test. The significance level was set at *p* = 0.05 and two-sided tests were used wherever possible. Tests were not corrected for multiple comparisons. Univariate odds ratios for predictors of significant coronary stenoses in coronary angiography were calculated using a logistic regression approach. Due to higher statistical power of percentile ranking, case–control matching was omitted. All statistical analyses were performed using the R Language and Environment for Statistical Computing, version 3.1.2 (R Foundation, Vienna, Austria).

## Results

### Demographic and clinical data

**Fig. 1:**
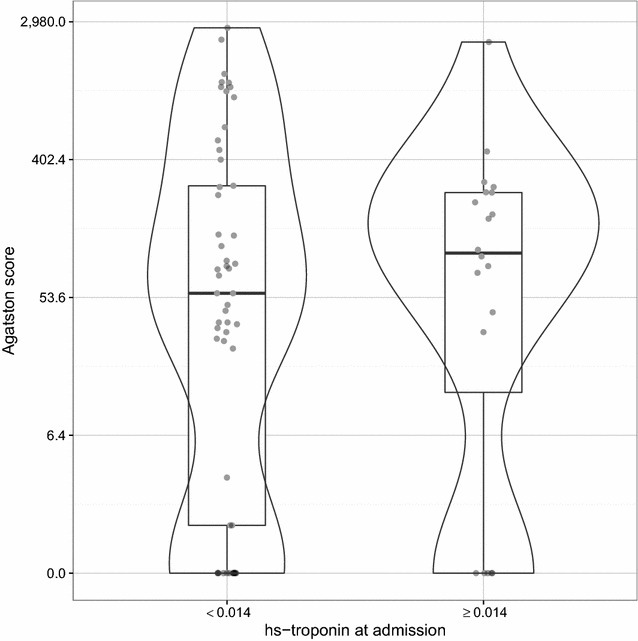
Agatston scores according to hs-troponin at admission. Distribution of Agatston scores, stratified by hs-troponin at admission (negative test, < 0.014 ng/ml; positive test, ≥ 0.014 ng/ml). Gray dots represent values of individual patients, plots are the smoothed estimated distributions of Agatston scores overlaid with Box plots. Scores are scaled according to *y* = log(Agatston + 1). *hs* high sensitive

A total of 618 patients were screened for study participation. 73 patients (59% female) patients fulfilled the inclusion criteria. Before CCT assessment, 24 patients or a proportion of 32% of the patients received treadmill testing first. In all of those cases, the results were judged as nondiagnostic during their index stay in the CPU. There was no additional noninvasive imaging (e.g., magnetic resonance imaging) other than transthoracic or transesophageal echocardiography. Median age did not differ significantly between female [73 (65–79) years] and male subjects [66 (56–74) years, *p* = 0.10]. Median heart rate at the time of CCT in sinus rhythm was 68 (59–74) bpm. Subjects with an Agatston score of 0 were significantly younger [62 (57–69) vs. 74 (66–79) years, *p* = 0.001] and tended to exhibit lower systolic blood pressure values than subjects with a score > 0 [125 (116–130) vs. 130 (125–150) mmHg, *p* = 0.061]. Baseline characteristics are summarized in Table 1. Typical symptoms at admission included atypical angina pectoris (100% by inclusion criteria), palpitations (78% in patients with an Agatston score of 0 vs. 82% otherwise, *p* = 0.74), dyspnea (33% vs. 62%, *p* = 0.055) and/or agitation (33% vs. 16%, *p* = 0.18) without any significant difference between those with and without CAC burden except for a trend to dyspnea in patients with coronary calcification. Further, there was no significant difference in conventional risk factors, left ventricular function or degree of mitral regurgitation with respect to presence or absence of CAC or relevant calcifying CAD as defined by an Agatston score > 75th percentile (Table 2). In addition, there was no significant difference for conventional risk factors between patients with compared to patients without relevant stenosis (Table 3). As displayed in Fig. 1, Agatston scores at admission were nonsignificantly higher in individuals with higher high sensitive- (hs-) troponin T values (all by definition not diagnostic for myocardial infarction, *p* = 0.8) [16]. When plotting CS categories (0–400, 400–1000, > 1000) against hs-troponin T, the highest values were found in patients with no or minimal CS (*p* = 0.38). Whereas hs-troponin values tend to be higher in patients in the lower CS category, no cutoff hs-troponin T value can be defined excluding high CS.

**Table 1:** Demographic variables, conventional risk factors, and echocardiographic data

	All patients (*n* = 73)	Agatston score			> 50th percentile (*n* = 22 of 44)
		0 (*n* = 18)	> 0 (*n* = 55)	*p* value	
Demographics					
Age	71 (62–78)	62 (57–69)	74 (66–79)	0.001	65 (59–69)
Female	43 (59%)	11 (61%)	32 (58%)	1	10 (45%)
History					
Smoking	6 (8%)	2 (11%)	4 (7%)	0.63	3 (14%)
Art. hypertension	57 (78%)	13 (72%)	44 (80%)	0.52	14 (64%)
Fam. history	22 (30%)	8 (44%)	14 (25%)	0.15	6 (27%)
HLP	38 (52%)	9 (50%)	29 (53%)	0.79	12 (55%)
DM	4 (5%)	1 (6%)	3 (5%)	1	1 (5%)
Measurements					
BMI	27 (25–30)	28 (26–30)	27 (24–30)	0.28	27 (25–30)
HR (at admission)	120 (91–135)	122 (106–139)	115 (90–134)	0.62	124 (97–130)
BP systolic	130 (120–150)	125 (116–130)	130 (125–150)	0.061	130 (116–140)
BP diastolic	80 (75–90)	80 (76–85)	80 (75–90)	0.91	80 (71–90)
HR (at CCT imaging)	68 (59–74)	70 (60–77)	67 (58–72)	0.25	69 (60–72)
CHA_2_DS_2_-VASc	3 (2–4)	2 (1–3)	3 (2–4)	0.11	2 (1–2)
Mitral regurgitation				0.17	
None	31 (42%)	9 (50%)	22 (40%)	n/a	8 (36%)
I°	34 (47%)	7 (39%)	27 (49%)	n/a	12 (55%)
I–II°	3 (4%)	2 (11%)	1 (2%)	n/a	1 (5%)
II°	5 (7%)	0 (0%)	5 (9%)	n/a	1 (5%)
Ejection fraction				0.28	
Normal	61 (84%)	16 (89%)	45 (82%)	n/a	19 (86%)
Slightly reduced	10 (14%)	1 (6%)	9 (16%)	n/a	3 (14%)
Moderately reduced	1 (1%)	0 (0%)	1 (2%)	n/a	0 (0%)

*BP* blood pressure, *CCT* cardiac computed tomography, *DM* diabetes mellitus, *BMI* body mass index, *HLP* hyperlipoproteinemia, *HR* heart rate

**Table 2:** Analysis of conventional risk factors and their association to relevant coronary calcification

	Agatston score		*p* value
	≤ 75th percentile (*n* = 40)	> 75th percentile (*n* = 20)	
Smoking	2 (5%)	3 (15%)	0.32
Art. hypertension	29 (72%)	16 (80%)	0.75
Family history of CAD	14 (35%)	6 (30%)	0.78
DM	1 (2%)	2 (10%)	0.26
Male sex	17 (42%)	11 (55%)	0.42
HLP	22 (58%)	11 (55%)	1
BMI	27 (25–30)	28 (25–30)	0.49
Number of conventional risk factors present	2 (1–3)	3 (2–3)	0.23

*CAD* coronary artery disease, *DM* diabetes mellitus, *BMI* body mass index, *HLP* hyperlipoproteinemia

**Table 3:** Evaluation of conventional risk factors and their impact on the need of coronary intervention in the subgroup of patients undergoing coronary angiography and showing relevant coronary calcification (> 75th percentile)

	Intervention		*p* value
	None (n = 8)	PCI/CABG (n = 4)	
Smoking	0 (0%)	0 (0%)	1
Art. hypertension	8 (100%)	4 (100%)	1
Family history of CAD	3 (38%)	0 (0%)	0.49
DM	1 (12%)	1 (25%)	1
Male sex	6 (75%)	2 (50%)	0.55
HLP	4 (50%)	2 (50%)	1
BMI	33 (28–37)	29 (28–30)	0.37
Number of conventional risk factors present	4 (3–4)	2 (2–3)	0.13

*PCI* percutaneous coronary intervention, *CABG* coronary artery bypass graft placement, *CAD* coronary artery disease, *DM* diabetes mellitus, *BMI* body mass index, *HLP* hyperlipoproteinemia

### CS: amount and distribution of CAC burden

**Fig. 2:**
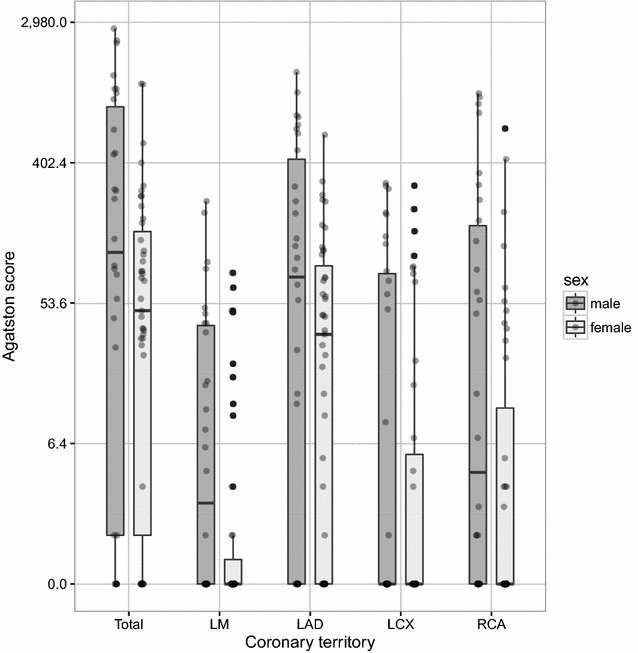
Local distribution of Agatston scores according to gender in different coronary artery territories. Box plot of Agatston scores by coronary territory. Scores are scaled according to *y* = log(Agatston + 1). Gray dots represent individual scores of each patient. *LM* left main, *LAD* left anterior descendent, *LCX* left circumflex artery, *RCA* right coronary artery

**Fig. 3:**
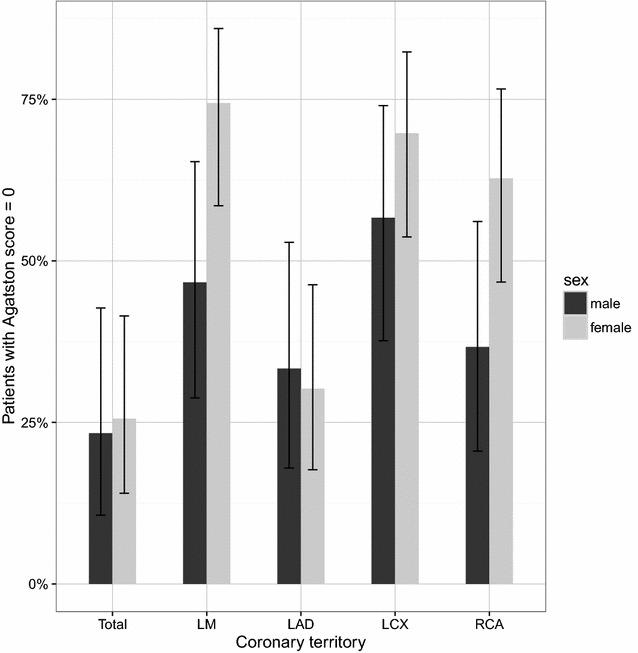
Proportion of patients without any relevant coronary artery calcification (Agatston score = 0) in the respective territory, stratified by gender. Error bars represent 95% confidence intervals. *LM* left main, *LAD* left anterior descendent, *LCX* left circumflex artery, *RCA* right coronary artery

Agatston scores ranged between 0 and 2726 with a median of 77 and an IQR of 1–270. When analyzed per territory, the highest Agatston scores were found in the LAD, and the median score in all other territories was 0 [LM: 0 (0–11); LAD: 44 (0–136), LCX: 0 (0–49); RCA: 0 (0–48), respectively]. Detailed data on gender-specific CAC burden per territory are given in Fig. 2 [scaled using the transformation *f*(*x*) = log(*x* + 1)]. In all territories, male subjects displayed higher Agatston scores. When comparing zero CS scores to positive CS scores, 23% of males (*n* = 7) and 25% of females (*n* = 11) had no CAC (Fig. 3). For those patients with positive CS scores, our study population shows a relative accordance to reference percentile distribution.

### CTA: obstructive lesions

Computed tomography angiography was performed in 34 patients (51% of females, 60% of males). In two cases, we performed retrospective gating due to significant supraventricular extrasystole, the remaining underwent prospective gating. All CTAs were judged as diagnostic and 136 vessels and 357 segments were analyzed in total. Significant stenoses were found in four patients or 12% of the CTAs (male: *n* = 0, female: *n* = 4). Stenotic vessels comprised LAD (*n* = 2) or RCA (*n* = 2) only.

### Decision for an invasive coronary angiogram: positive CTA versus high CS

A total of 16 subjects underwent invasive angiography. The decision to perform coronary catheterization is assumed to be made because of a positive CTA in 4 cases, and because of high CS in 12 cases. Invasive angiography revealed significant coronary artery stenosis in 5 subjects and only mild to intermediate stenosis in the remaining 11 subjects. Two individuals underwent percutaneous intervention (one patient with positive CTA, one patient with coronary angiography due to high CS), and in 3 cases an aortocoronary bypass graft placement was performed (one patient with positive CTA, and two patients with coronary angiography due to high CS). In half of the patients in whom the decision to perform coronary angiography was presumably made because of a positive CTA (*n* = 4), significant stenosis was confirmed by coronary angiography. In those subjects with an invasive regimen, median Agatston scores were high [1041 (374–1232)] with the highest values in the LAD [394 (223–660)]. Patients with revascularization exhibited higher CS within the LM territory [83 (67–97)] compared to those without [11 (2–38)]. On univariate analysis, relevant CS, defined as an Agatston above average (> 50th percentile), did not serve as an independent predictor for coronary stenosis during coronary angiography (odds ratio: 1.47, confidence interval 0.71–3.03; *p* = 0.32).

## Discussion

According to CPU registry data, CCT is not commonly used in German CPUs. Only about 5% of all-comers received CCT following admittance to a CPU, mostly as a rule-out approach in the majority of cases. Importantly, however, even this small number of patients receiving CCT helped to prevent unnecessary invasive catheterization procedures, since patients with CCT underwent significantly less often coronary angiography or revascularization [9]. Nonetheless, target CPU subsets have to be identified that may profit most from noninvasive imaging procedures. It has been shown that CCT allows to accurately rule out coronary stenoses in patients with an intermediate pretest likelihood for CAD [17]. Our current study was conducted to evaluate the impact of CCT for patients presenting to the CPU with symptomatic, non-ACS, new-onset AF and an intermediate pretest probability for CAD. The main findings are: (i) CAD could be virtually excluded in 25% of all patients by zero CS, (ii) CS scores above the median were present in about 50%, reflecting a good accordance between the study and the reference percentile distribution, (iii) 22% of all patients could be stratified as “at risk” due to a positive CTA or high (CS > 400) or very high (CS > 1000) CAC burden, and (iv) 7% of the cohort subjects underwent a coronary intervention, suggesting a potential role of AF as trigger of ischemia. Thus, the use of additional CCT during CPU work-up was able to identify relevant atherosclerotic comorbidity in up to one quarter of the patients as compared to standard work-up. Nonetheless, relevant CS, defined as an Agatston above the median (> 50th percentile), was not a predictor for coronary stenosis during coronary angiography in our study population; this effect could have been caused by small sample size, however.

Although sample size was too small for statistical subgroup analyses, median CS scores tended to be higher in males. These gender-specific scores were comparable to those found in AF subjects with known cardiovascular disease or treated risk factors in the unselected general population of the Heinz Nixdorf Recall study. In their study, Möhlenkamp et al. [18] evaluated the association of CAC burden assessed by CCT with resting ECG abnormalities. Keeping in mind that their study population was nearly devoid of subjects with AF and known cardiovascular disease or treated risk factors, they found that a CAC score > 75th percentile was more prevalent in AF subjects. These results are in conflict with ours, which show a good accordance between the percentiles of our study population and the reference percentile distribution resulting from the Heinz Nixdorf Recall study. Previous literature focusing on the relationship between atrial tissue ischemia, CAD and the coronary artery tree remains inconsistent [19–21]. In our study, we found the highest overall Agatston scores in the LAD in patients undergoing coronary intervention/revascularization, and calcification of the LM was more often present in patients with significant stenosis. Different as expected and in line with previous results, elevated hs-troponin levels neither exhibited a certain cutoff level to discriminate between those with low or high calcium burden, nor served as a marker for identifying patients at risk as the highest amounts were even found within the zero CS or low CS groups, together arguing against the hypothesis of a lower ischemic tolerance in high CS patients in our subset [22].

High (> 400) or very high (> 1000) CS scores were present in nearly a quarter of the study population, which appears to be higher than those anticipated and higher than those previously reported within a population-based approach [23]. Of note, one of the conventional risk factors was able to significantly differentiate between those with and without relevant calcification, and conventional risk factors also failed to distinguish between those with and without relevant stenosis. In our study, we found that 12 out of 73 patients received coronary angiography due to high CS. Of those, three patients (equaling 25%) needed coronary intervention. However, as the study was designed as a retrospective observational study, we do not have invasive controls of CCT findings by coronary angiography as gold standard. Therefore, the definite role of different amounts of coronary calcification remains open. On the other hand, up to the current literature, CTA is supposed to be more sensitive than any other available noninvasive imaging technique, while the recommendation in patients with low-to-intermediate pretest probability for CAD stems from a high negative predictive value (99–100%) [24, 25]. In our study, additional CTA was performed in 47% of all patients and judged positive in 12%. Even though the positive predictive value of CCT/CTA was limited, as much as one-third of patients triaged to further invasive diagnostics following CCT displayed relevant stenosis afterward. This percentage is unexpectedly high compared with the current data from a CPU registry, demonstrating a low rate of coronary intervention in “real” troponin-negative ACS patients even including high-risk patients of 18%, although only about 7% exhibited AF at admission [26]. Keeping in mind that all patients were triaged as non-ACS patients at the time of admission by the CPU physician and, therefore, that the study cohort mainly consisted of non-target CPU patients, CCT is thought to add crucial diagnostic information in the diagnostic work-flow even in patients presenting with atypical chest pain, the first diagnosed AF, and intermediate pretest probability for CAD admitted to a CPU for their atypical symptoms, yet on-top of the main CPU subset presenting with typical ACS. Whether we were able to visualize only subclinical atherosclerosis or whether CCT was able to detect CAD as an ischemic trigger of the new-onset AF remains speculative. However, we anticipate that using standard protocols, most of the patients declared as at risk following CCT would have been simply discharged following cardioversion and might have returned shortly after as “real” CPU patients with coronary event.

Besides those “positive” patients, 25% of our patients had CS of zero. The diagnostic value of zero CS within patients presenting with acute chest pain to the emergency department has been previously discussed [27–29]. In summary, the prevalence of significant CAD seems to be low and the negative predictive value high. Even though, because of the design of our study, coronary angiography was not performed as a gold standard in patients with zero CS and thus validation is biased, based on the available studies, we still propose that a CS of zero may also be used as a gatekeeper prior to further diagnostic steps in our commonly encountered subgroup.

In addition to the designated diagnostic value of CCT to prevent unnecessary invasive catheterization procedures, thus avoiding risk to the patient and saving costs in the healthcare system, as well as the value to indicate those with a high risk of obstructive CAD, there still remained slightly more than a half within the “gray zone” of low-to-average CAC burden or high CAC burden but nonobstructive CAD. But even in this part of the population, patients may benefit from atherosclerotic disease quantification in terms of more intense risk factor modification [8, 30]. On the other hand, ruling out relevant CAD may also be helpful in choosing potential drugs for antiarrhythmic therapy, particularly when the use of class I antiarrhythmic drugs, such as flecainide, is considered [31].

### Study limitations

This study is a retrospective, single-center, observational project on a small cohort with inherent limitations and may be underpowered due to the small sample size. In this respect, the study lacks comparison to results of CCT in a control group of patients with atypical chest pain and an intermediate pretest probability but without AF admitted to CPU. Small sample size forced a descriptive approach in some subgroup analyses. Exclusion of patients with relevant kidney failure causes a systematic bias of patients at risk for CAD, additionally—even though by clinical pathways, all patients with the new-onset AF and atypical chest pain and low-to-intermediate pretest probability should be included—a certain selection bias by retrospectively analyzing CCT data only cannot be completely excluded. The fact that only subjects with high CS or obstruction in CTA underwent coronary angiography represents a major bias. This means that CAD could not be completely ruled out in the low CS and nonobstructive CTA group. However, the decision for invasive angiography was made by the cardiac interventionalist on the case based on clinical decision pathways, and not by randomization. In addition, we are aware of the fact that a zero CS alone cannot completely rule out CAD, but is only associated with a very low probability and good prognosis [32]. Based on our inclusion criteria, our cohort merely consisted of patients with the new-onset AF with image acquisition following cardioversion. Thus, our findings cannot be transferred to other AF forms, especially permanent AF. Nonetheless, according to analyses using new generation scanner, it appears feasible to extensive use and scientific efforts to these groups, too [33, 34]. Not the least, controversies with regard to the recent PROMISE data on nonsuperiority of CCT against functional stress testing showing higher rates of coronary revascularization following CCT without benefit during long-term outcome have to be considered [35].

### Conclusions

In our study population, CCT was able to virtually exclude relevant CAD in about one quarter and detect relevant CAD in about another quarter of the subjects, putting those patients on a higher risk level and accelerating standard diagnostics. As much as 31% of those patients declared as at risk equaling 7% of the total study population needed coronary intervention. For the rest, analysis of CAC burden may be useful to guide risk factor modification efforts or to aid in the choice of a drug class for antiarrhythmic therapy. Thus, our data underline the benefit of CCT within the CPU concept at least in this selected but commonly encountered non-target CPU patient population by having a strong impact on patients’ management in terms of improved risk stratification, risk factor modulation, and selection for additional interventional diagnostics. Nevertheless, as in our small cohort CAC burden exceeding the 50th percentile statistically has not been proven to be an independent predictor of coronary stenosis, our suggestion should be evaluated in larger studies on a prospective and case-controlled basis.

## Abbreviations

AF: atrial fibrillation
CAC: coronary artery calcification
CAD: coronary artery disease
CCT: cardiac computed tomography
CPU: chest pain unit
CS: calcium score
CTA: computed tomography angiography
IQR: interquartile range
HNR: Heinz Nixdorf Recall
hs-troponin: high sensitive troponin
LAD: left anterior descendent
LCX: left circumflex artery
LM: left main
RCA: right coronary artery
